# Olfactory Dysfunction as a Clinical Marker of Early Glymphatic Failure in Neurodegenerative Diseases

**DOI:** 10.3390/diagnostics15060719

**Published:** 2025-03-13

**Authors:** Gonzalo Sánchez-Benavides, Alex Iranzo, Oriol Grau-Rivera, Darly Milena Giraldo, Mariateresa Buongiorno

**Affiliations:** 1 Barcelonaβeta Brain Research Center (BBRC), Pasqual Maragall Foundation, 08005 Barcelona, Spain; 2 Hospital del Mar Research Institute, 08003 Barcelona, Spain; gsanchezb@barcelonabeta.org (G.S.-B.); ograu@barcelonabeta.org (O.G.-R.); 3 Centro de Investigación Biomédica en Red de Fragilidad y Envejecimiento Saludable (CIBERFES), 28029 Madrid, Spain; 4 Sleep Disorders Center, Neurology Service, Hospital Clínic Universitari de Barcelona, University of Barcelona, 08036 Barcelona, Spain; airanzo@clinic.cat; 5 Institut d’Investigacions Biomèdiques August Pi i Sunyer (IDIBAPS), 08036 Barcelona, Spain; 6 Centro de Investigación Biomédica en Red Sobre Enfermedades Neurodegenerativas, 28031 Madrid, Spain; 7 Neurology Department, Vall d’Hebron University Hospital, 08035 Barcelona, Spain; darlymilena.giraldo@vallhebron.cat; 8 Neurovascular Diseases Research Group, Vall d’Hebron Research Institute, 08035 Barcelona, Spain

**Keywords:** neurodegenerative diseases, olfactory dysfunction, glymphatic system, Alzheimer’s disease, Parkinson’s disease, Lewy body diseases

## Abstract

An abnormal accumulation of misfolded proteins is a common feature shared by most neurodegenerative disorders. Olfactory dysfunction (OD) is common in the elderly population and is present in 90% of patients with Alzheimer’s or Parkinson’s disease, usually preceding the cognitive and motor symptoms onset by several years. Early Aβ, tau, and α-synuclein protein aggregates deposit in brain structures involved in odor processing (olfactory bulb and tract, piriform cortex, amygdala, entorhinal cortex, and hippocampus) and seem to underly OD. The glymphatic system is a glial-associated fluid transport system that facilitates the movement of brain fluids and removes brain waste during specific sleep stages. Notably, the glymphatic system became less functional in aging and it is impaired in several conditions, including neurodegenerative diseases. As the nasal pathway has been recently described as the main outflow exit of cerebrospinal fluid and solutes, we hypothesized that OD may indeed be a clinical marker of early glymphatic dysfunction through abnormal accumulation of pathological proteins in olfactory structures. This effect may be more pronounced in peri- and postmenopausal women due to the well-documented impact of estrogen loss on the locus coeruleus, which may disrupt multiple mechanisms involved in glymphatic clearance. If this hypothesis is confirmed, olfactory dysfunction might be considered as a clinical proxy of glymphatic failure in neurodegenerative diseases.

## 1. Introduction

An abnormal accumulation of misfolded proteins, such as amyloid-β (Aβ) and tau in Alzheimer’s disease (AD) and α-synuclein in the spectrum of Lewy body diseases (LBD), is a common feature shared by most neurodegenerative disorders. In 2012, a new functional system devoted to the clearance of solutes from the brain -the glymphatic system was described- [1] and, in the few last years, a growing body of evidence has suggested that the failure in protein clearance by this system may play a capital role in the etiology and progression of neurodegenerative diseases [2]. Besides, olfactory dysfunction (OD) is common in the early stages of AD and LBD and has been related to initial neuropathological changes in the olfactory bulb and related brain structures [3]. As the nasal pathway has been recently described as the main outflow pathway of cerebrospinal fluid (CSF) and solutes [4], we hypothesized that OD may indeed be a clinical marker of early glymphatic dysfunction that entails the abnormal accumulation of pathological proteins in olfactory structures. In the present paper, we briefly review the evidence of OD in neurodegenerative diseases, focusing on early and preclinical stages, and we introduce the hypothesis that OD may be a clinical marker of early glymphatic system failure, aiming to foster new research on the topic.

## 2. Olfactory Dysfunction in Neurodegenerative Diseases

It is well-established that olfactory ability decreases with age, significantly affecting safety, nutrition, and the physical and mental well-being of the elderly [5]. Considering pathological thresholds of smell loss, the observed prevalence of anosmia above 65 years old is high, around 13–14% [6,7], increasing up to >60% above 80 years old in some studies [8]. OD also correlates with cognitive abilities [9,10], and its presence doubles the odds of concurrent cognitive impairment [7]. Indeed, OD is present in 90% of patients with AD or Parkinson’s disease (PD) [11], preceding the cognitive and motor symptoms onset by several years [12,13]. The main brain structures involved in odor processing (olfactory bulb and tract, piriform cortex, amygdala, entorhinal cortex, and hippocampus) are compromised in such diseases and seem to underly OD [14]. In the following paragraphs, we provide a brief review on the association between olfactory dysfunction and early AD and LBD.

### 2.1. OD in Preclinical Alzheimer’s Disease

Alzheimer’s disease is currently conceptualized as a clinical–biological entity, in which neuropathology slowly emerges up to two decades before the onset of cognitive symptoms [15]. The period in which there is pathophysiological evidence of AD-related changes (namely abnormal Aβ and tau accumulation) in the absence of cognitive deterioration is referred to as the preclinical AD stage. However, in recent years, accumulating evidence shows that some subtle cognitive deficits can be observed when challenging memory paradigms or intraindividual change are considered [16,17]. In parallel to this sub-threshold cognitive decline, olfactory disturbances also emerge. It is known that odor identification is already impaired in cognitively normal *APOE-ε4* carriers, the main risk gene for sporadic AD [18,19], and that those individuals harboring Aβ pathology with anosmia are at increased risk of accelerated cognitive decline [20]. Indeed, Palta et al. (2018) found that poor olfactory function in late life was related with greater prior 20-year cognitive decline [21], and two large longitudinal studies observed hazard ratios around two for the incidence of mild cognitive impairment (MCI) at 3.5 years [22] and dementia at 12 years [23]. Another recent study showed that those individuals who will develop MCI had a faster decline in olfactory identification that started five years preceding MCI diagnosis, and such decline accelerates three years before dementia diagnosis [24]. Regarding the relationship between OD and AD biomarkers, a small meta-analysis on 9 studies including cognitively unimpaired (CU), MCI, and AD patients concluded that the association between odor identification and Aβ and tau burden was negligible [25]. However, when the analyses were restricted to the CU (5 studies), there was an association between olfaction and Aβ-PET deposition [25]. As suggested by the authors, the fact that Aβ accumulation reaches a plateau in mild to moderate AD stages may underlie the negative findings in the full spectrum of the disease. OD has been related to lower hippocampal volumes and cortical thinning in AD-vulnerable regions (see [26] for a review)and more recent studies also observed that anosmia was significantly associated with higher plasma t-tau and neurofilament-light levels in community dwelling individuals after the exclusion of those with MCI [27]. Taken together, all these results provide compelling evidence on the very early compromise of olfactory function in the preclinical stage of AD.

### 2.2. OD in Prodromal Lewy Body Diseases

The cluster of disorders characterized by pathological deposition of α-synuclein aggregates within the central and peripheral nervous system are collectively labelled as α-synucleinopathies. While in PD and dementia with Lewy bodies (DLB)—both constituting the group called Lewy body diseases (LBD)—α-synuclein aggregates are intraneuronal, multiple system atrophy (MSA) is characterized by oligodendroglial cytoplasmic inclusions. Rapid eye movement (REM) sleep behavior disorder (RBD) is a specific manifestation of the prodromal stage of α-synucleinopathies [28]. Follow-up studies have shown that more than 80% of patients with RBD will develop a Lewy body disease—either PD or DLB—or, less commonly, MSA [29,30]. OD is almost universal in established PD, but it is also common in patients with isolated RBD, representing a predictor of short-term phenoconversion [31,32,33]. OD in LBD is likely related to Lewy pathology and neuronal cell loss in the olfactory bulbs, tracts, and piriform cortex, and according to the Braak staging, the olfactory bulbs may be an initial site of α-synuclein aggregation [34]. The presence of OD in prodromal PD has important implications for disease pathogenesis and progression. It has been suggested that PD originates in peripheral sites, such as the olfactory system and gastrointestinal tract, probably by a neurotropic pathogen, before spreading to the central nervous system via interconnected pathways, including the vagus nerve and olfactory tracts [35,36]. Increasing evidence supports the gut-to-brain hypothesis in an elevated proportion of PD patients. Pathological α-synuclein has been detected in gastrointestinal tissues before motor symptoms appear [37], and gut dysbiosis may contribute to neuroinflammation and α-synuclein misfolding [38,39]. The gut-first pattern is tightly related to the presence of RBD, and it has been reported that patients with idiopathic RBD display OD and neuronal dysfunction in the peripheral nervous system and locus coeruleus equivalent to PD patients but still normal nigrostriatal dopaminergic innervation [40]. We previously hypothesized that early dysautonomia associated with the gut-first pattern may impair glymphatic function due to sleep-related blood pressure abnormalities [41], as vascular motion is a key driver of glymphatic clearance (see Section 3). Notably, OD appears to be tightly linked to these early changes, suggesting a potential mechanistic connection between olfactory impairment, autonomic dysfunction, and impaired glymphatic clearance in the gut-first trajectory of PD.

## 3. Glymphatic System and Its Failure in Neurodegenerative Diseases

In peripheral tissues, capillaries enable a steady flow of plasma ultrafiltrate, which distributes nutrients, energy metabolites, and signaling molecules. This fluid mixes with interstitial fluid (ISF) within tissues, and excess fluid is removed via the lymphatic system [42]. In the brain, however, plasma ultrafiltrate inflow is restricted by the blood–brain barrier (BBB). The brain produces its own fluid, the CSF, and was historically thought to lack lymphatic vessels. Given the brain’s high metabolic activity, the absence of lymphatic drainage posed a challenge to understanding how it maintains homeostasis.

In 2012, a glial-associated fluid transport system, named the glymphatic system, was identified in the brain [1]. The glymphatic system facilitates the movement of CSF and ISF through three compartments. The first compartment involves CSF inflow into periarterial spaces around arteries that penetrate the brain parenchyma. In the second compartment, CSF and ISF mix within the brain’s interstitial spaces, aided by aquaporin-4 (AQP4) water channels on astrocytic endfeet lining periarterial spaces [1,43]. These astrocytic endfeet are polarized toward the basal lamina, facilitating fluid movement along the glymphatic pathway [44]. Waste products in the ISF are transported toward perivascular spaces on the venous side and cleared from the central nervous system via a combination of diffusion and convection [45]. The third compartment, glymphatic efflux, involves drainage of ISF into perivenous spaces. Here, metabolic and neurotoxic wastes exit via meningeal and cervical lymphatic vessels, as well as along cranial and spinal nerves. Glymphatic flow is driven by arterial pressure waves from cardiac pulsation, respiration, and slow vasomotion [46,47] and it is mainly active during sleep [48]. Downstream of the glymphatic system lies the meningeal lymphatic system, which acts as the fourth compartment of brain fluid transport [49]. These lymphatic vessels in the meninges remove waste, signaling molecules, and solutes from the CNS transported by the glymphatic system.

The glymphatic system becomes less functional in aging and is impaired in several conditions, including head trauma, subarachnoid hemorrhage, and neurodegenerative diseases. In 2020, Nedergaard and Goldman proposed that glymphatic failure may represent a final common pathway to dementia [2]. Although it has been thought that misfolded proteins, such as Aβ and tau in AD, or α-synuclein in PD spread across synaptically connected regions [50], evidence shows that their spread occurs in both forward and backward directions. Based on this observation, their hypothesis is that the aggregates move through extracellular spaces, and their accumulation is linked to reduced glymphatic flow with aging. The way protein aggregates spread in the brain is different in each disease but seems to match the pathways of glymphatic CSF flow. In AD, Aβ first deposits in the frontal and parietal lobes, spreading later to the hippocampus and temporal cortex. However, cognitive decline is more related to tau pathology, which begins in the entorhinal cortex and later involves the hippocampus and neocortex [51]. In PD, α-synuclein aggregates start in the brainstem and olfactory bulb, spreading to limbic areas and, eventually, to the cortex. MRI studies using intrathecal gadobutrol show that impaired glymphatic flow traps substances in the same regions where aggregates form [52,53], suggesting that reduced clearance, rather than synaptic connectivity, could explain the patterns of protein spread [2].

Aging is the main risk factor for neurodegenerative diseases and also causes poor sleep quality, particularly a reduction in deep non-REM sleep, which is crucial for waste clearance [54]. This worsening of sleep may lead to higher protein accumulation and faster disease progression. Interestingly, sleep disturbances in PD often occur years before motor symptoms appear [55], and these disturbances may also play a role in the spread of aggregates. As previously introduced, we have proposed a model suggesting that sleep disorders, combined with dysautonomia—which disrupts the vascular motion driving of glymphatic influx—may lead to a critical failure of glymphatic function. This mechanism could help to explain the heterogeneity observed in the clinical progression of α-synucleinopathies [41].

## 4. Menopause, Olfactory Dysfunction, and Glymphatic Failure

In recent years, some researchers have proposed that the downstream effects of menopause may contribute to the disproportionate vulnerability of females to AD compared to males, with an estimated female-to-male ratio of approximately two to one [56]. Luckey et al. have proposed a hypothesis to explain the increased vulnerability of females highlighting the differential and detrimental effects of estrogen loss in the locus coeruleus-norepinephrine (LC-NA) system in women [57]. Estrogens influence LC-NA regulation through nuclear estrogen receptors (ERs) and the cell-membrane ER subtype G protein-coupled estrogen receptor 1, which are widely distributed throughout the LC [58]. Given that menopause leads to a rapid decline in ovarian sex-hormones during midlife, the authors hypothesize that estrogen loss may drive the deterioration of the female LC-NA system, ultimately increasing the risk of developing AD. However, the precise mechanisms by which LC-NA dysregulation increases the risk of developing AD remain unclear. Based on converging evidence, we hypothesize that LC degeneration contributes to AD risk by impairing glymphatic function. It is well established that LC activity regulates circadian rhythms and sleep phases and also plays a critical role in modulating the neurovascular unit (NVU) through noradrenergic fibers that innervate intraparenchymal arterioles and capillaries. At the capillary level, LC-NA terminals directly interact with the NVU, specifically targeting astrocyte end-feet and pericytes [59]. Together, these components form the main functional unit of the glymphatic system. Sleep disturbances are well known to be associated with AD and have been identified as a risk factor for dementia [60,61]. Notably, women appear to be disproportionately affected by sleep problems compared to men, with sleep difficulties often emerging during the menopausal transition [62], likely due to the aforementioned loss of estrogen’s regulatory influence over LC-NA.

Beyond its role in sleep regulation, estrogen has also been implicated in olfactory function. Post-menopausal women displayed increased rates of OD [63], and hormone replacement therapy improves olfactory function [64]. Experimental evidence also suggests that estradiol prevents olfactory dysfunction induced by Aβ toxicity in the hippocampus [65]. Supporting this, a recent fMRI study demonstrated reduced activation of the primary olfactory cortex and hippocampus in women compared to men between the ages of 50 and 60 [66]. Building on this, Ekanayake et al. hypothesized that menopausal women experiencing poor sleep may also suffer from impaired glymphatic function, leading to inefficient clearance of toxins and metabolic waste. This dysfunction could trigger inflammation in the olfactory mucosa, olfactory bulb, and entorhinal cortex—brain regions known to be among the first affected in AD—potentially contributing to preclinical olfactory decline before neurodegeneration spreads to other regions [67]. An additional effect of menopause in women is the increase in blood pressure (BP) and the more frequent occurrence of abnormal BP circadian variability [68]. Vascular pulsatility is a key driver of glymphatic function, and both hypertension [47] and the loss of the physiological BP dip during sleep may impair clearance. Indeed, abnormal sleep BP patterns have been associated with an increased risk of AD [69] and Aβ deposition [70]. Therefore, vascular changes in postmenopausal women may further contribute to glymphatic dysfunction.

A potential challenge to our hypothesis of menopause-related glymphatic failure within a broader spectrum of protein-accumulating diseases is the reported higher prevalence of PD in men compared to women [71]. However, recent findings suggest that this sex difference may be smaller than previously assumed [72]. Notably, when accounting for age, the male-to-female ratio peaks in the fifties—possibly reflecting the residual protective effects of estrogens—but becomes non-significant thereafter [73].

## 5. Olfactory Dysfunction as a Clinical Marker of Glymphatic Failure in Neurodegenerative Disease

We hypothesize that OD could be tightly associated with glymphatic dysfunction. This suggestion is based on the following physiological, anatomical, and chronological evidence.

**Figure 1:**
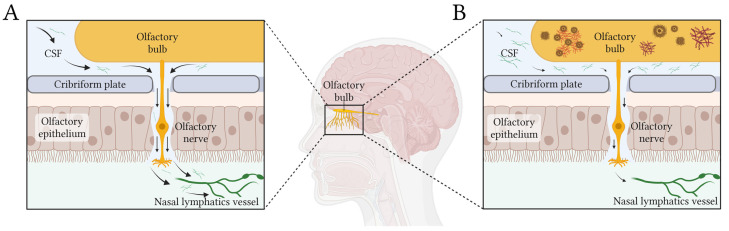
Simplified scheme of CSF egress through the periolfactory pathway. Black arrows show the CSF flow in healthy (**A**) and pathological (**B**) conditions. In panel (**B**), the diminished flow of CSF produced by glymphatic dysfunction would generate stagnation of solutes and abnormal accumulation of proteins in the olfactory bulb and related structures. Created in BioRender https://BioRender.com/l91w249.

(1) **Olfactory structures show early protein aggregation.** Early Aβ, tau and α-synuclein protein aggregates have been observed in the olfactory bulb, olfactory epithelium, piriform cortex, the glomerular layer, anterior olfactory nucleus, and olfactory tubercle [74,75,76]. In LBD, the olfactory bulbs are an initial site of α-synuclein aggregation [34]. Neuropil threads and neurofibrillary tangles of tau protein have been observed in the olfactory bulb and olfactory nerve in all cases of definite AD, as well as in many cases of probable AD, MCI, and even cognitively normal aging [77,78]. Moreover, in a healthy elderly population, primary age-related tauopathy typically concentrates in the medial temporal lobe and olfactory regions, including the olfactory bulb, transentorhinal region, and entorhinal cortex [79,80].
(2) **Nasal pathway is the main CSF egress from the brain.** CSF is primarily cleared along olfactory nerves that traverse the cribriform plate, draining into lymphatic vessels in the nasal mucosa [81]. De Leon and colleagues demonstrated, using dynamic PET, that, as in other mammals, human nasal turbinates are part of the CSF egress system [82]. They also observed that such clearance measures were 66% lower in AD patients. Using a different technique, serial MRI after an intrathecal contrast injection, Zhou et al. found concordant results [4]. They observed drainage of CSF to the turbinates via the cribriform plate along the olfactory nerve, this being the main egress pathway. Moreover, clearance function through the peri-olfactory inferior turbinate pathway was diminished with aging and was associated with cognitive function and reported sleep quality [4]. Figure 1 depicts the physiological CSF egress through the perineural olfactory pathway (Figure 1A) and the pathological protein aggregation associated with diminished clearance (Figure 1B).

(3) **Glymphatic failure seems to be a very early event in neurodegenerative diseases.** Recent evidence suggested that glymphatic function is affected very early in AD. Using diffusion tensor imaging (DTI) along perivascular spaces (ALPS), a neuroimaging proxy of glymphatic function, two large longitudinal studies found that the ALPS index becomes abnormal in individuals with subjective cognitive decline (SCD), before objective cognitive impairment can be detected [83,84]. Moreover, the lower ALPS index predicts accelerated Aβ PET burden and AD signature ROI thinning, higher risk of amyloid-positive transition, and faster cognitive decline [83]. Based on such evidence, Huang et al. proposed a hypothetical cascade model of pathological events in AD, in which reduction in the ALPS index would be the first biomarker to change. The evidence in LBD is similar. Using the DTI-ALPS index as well, Bae et al. found that glymphatic function was diminished in RBD patients and that the lower ALPS index predicted phenoconversion to PD [85]. Accordingly, they concluded that glymphatic impairment is presumed to start at the preclinical stage of PD.
(4) **Olfactory dysfunction is associated with loss of estrogen after menopause and parallels glymphatic-related events.** Olfactory dysfunction in postmenopausal women seems linked to estrogen loss, paralleling glymphatic dysfunction. Estrogen influences LC-NA regulation, sleep, and vascular function, all of which are critical for glymphatic clearance [59]. Sleep disturbances and abnormal BP patterns, both prevalent after menopause, further compromise glymphatic efficiency and may contribute to AD risk. Experimental and neuroimaging studies support estrogen’s role in olfactory function, suggesting that its decline may accelerate neurodegenerative processes.

## 6. Concluding Remarks

**Figure 2:**
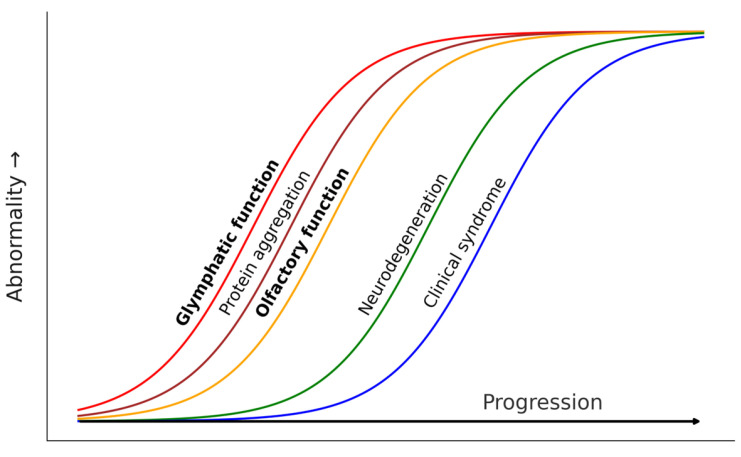
Theoretical progression model of pathological events in protein accumulation neurodegenerative diseases including glymphatic and olfactory dysfunction as early events concurrent to early protein aggregation.

Based on the evidence presented, we hypothesize that OD may be a clinical proxy of glymphatic dysfunction in neurodegenerative diseases. Our proposal relies on the chronological, physiological, and anatomical overlap of both phenomena. Although the idea that the disruption of CSF through the olfactory system may contribute to AD pathogenesis is not new [86] and olfactory impairment is well-established in LBD, this is the first time in which a close link between OD and glymphatic dysfunction is explicitly proposed. In Figure 2 we summarize our proposal, suggesting that glymphatic failure, protein aggregation in olfactory structures, and OD would concurrently emerge at the very early stages of neurodegenerative diseases. On top of genetic predisposition for such diseases, sleep disturbances, deleterious genetic variants of *APOE* and *AQP4*, circadian vascular abnormalities, lifestyle, and other unknown factors would modulate the emergence and progression of glymphatic failure, triggering the downstream processes.

This effect may be more pronounced in peri- and postmenopausal women due to the well-documented impact of estrogen loss on the locus coeruleus, which may disrupt multiple mechanisms involved in glymphatic function. If our hypothesis is proven true, olfactory performance could be used to identify individuals with emerging glymphatic dysfunction, select them for therapeutic trials targeting solute clearance, and monitor their efficacy.

## Data Availability

No new data related to this article were generated.
